# Excitation Energy Transfer Dynamics from Carotenoid to Bacteriochlorophyll *a* in the LH2 Complex of *Rhodobacter sphaeroides*: Insights from Reconstitution Experiments with Carotenoids and B800 Bacteriochlorophyll *a*

**DOI:** 10.3390/molecules30040814

**Published:** 2025-02-10

**Authors:** Chiasa Uragami, Marina Yoshida, Alastair T. Gardiner, Richard J. Cogdell, Hideki Hashimoto

**Affiliations:** 1 Department of Applied Chemistry for Environment, Graduate School of Science and Technology, Kwansei Gakuin University, 1 Gakuen-Uegahara, Sanda 669-1330, Japan; chiasa.uragami@kwansei.ac.jp (C.U.);; 2 Institute of Microbiology, Czech Academy of Sciences, 379 81 Trebon, Czech Republic; gardiner@alga.cz; 3 School of Molecular Biosciences, University of Glasgow, Glasgow G12 8QQ, UK; richard.cogdell@glasgow.ac.uk

**Keywords:** purple photosynthetic bacteria, light-harvesting, photoprotection, carotenoid, B800 bacteriochlorophyll *a*, reconstitution

## Abstract

Carotenoids are crucial for photosynthesis, playing key roles in light harvesting and photoprotection. In this study, spheroidene and bacteriochlorophyll *a* (Bchl *a*) were reconstituted into the chromatophores of the carotenoidless mutant *Rhodobacter sphaeroides* R26.1, resulting in the preparation of high-quality LH2 complexes. Global and target analyses of transient absorption data revealed that incorporating B800 Bchl *a* significantly enhances excitation energy transfer (EET) efficiency from carotenoids to Bchl *a*. EET predominantly occurs from the carotenoid S_2_ state, with additional pathways from the S_1_ state observed in native LH2. Unique relaxation dynamics were identified, including the generation of the carotenoid S* state in reconstituted LH2 with both spheroidene and B800 Bchl *a* and the formation of the carotenoid T_1_ state in reconstituted LH2. These findings underscore the critical influence of pigment composition and spatial organization on energy transfer mechanisms. They provide valuable insights into the molecular interplay that governs excitation energy transfer in photosynthetic light-harvesting systems.

## 1. Introduction

Plants and photosynthetic bacteria rely on pigments like carotenoids and chlorophyll for photosynthesis. Carotenoids absorb visible light (400–600 nm), transferring energy to chlorophyll and bacteriochlorophyll (Bchl) while protecting cells by dissipating excess energy [1,2,3]. While the fundamental energy conversion systems in plants and photosynthetic bacteria share similarities, the bacterial systems are structurally simpler. In these bacteria, pigments are intricately organized within photosynthetic membranes at the nanoscale, ensuring efficient light energy conversion. Among photosynthetic bacteria, purple photosynthetic bacteria have garnered significant attention due to their relatively simple structure, making them a model system for studying the basic mechanisms of photosynthesis [4,5].

Purple photosynthetic bacteria are rich in light-harvesting (LH) complexes, which efficiently capture diffuse sunlight [6]. These bacteria have two types of antenna complexes: the core LH1 and peripheral LH2, both composed of carotenoids and Bchl *a* within α-β helix proteins [5]. LH1 contains 15 or 16 subunits [3,7,8,9,10], while LH2 has 7–9 subunits [11,12,13,14], each with three Bchl *a* molecules and one carotenoid. Two Bchl *a* molecules form a dimer, B850 Bchl *a*, while the third is B800 Bchl *a* monomer. The structural organization of LH2 is illustrated in Figure S1A in Supplementary Materials [14]. A carotenoidless mutant strain, *Rhodobacter* (*Rba.*) *sphaeroides* R26.1, lacks carotenoids and B800 Bchl *a* but can grow under anaerobic light conditions [3,15].

Light harvesting is critical for the efficiency and functionality of photosynthesis, serving as the gateway for light energy entering the cell. The captured energy is transferred with extraordinary speed—on the order of femto-seconds (10^−15^ s) to picoseconds (10^−12^ s)—and is delivered to the reaction center (RC) with nearly 100% efficiency, where it triggers charge separation [16]. In this process, carotenoids play two key roles: they act as light-harvesting pigments by absorbing and transferring energy to chlorophyll and serve as photoprotective agents, preventing damage from excess light. This protection involves singlet oxygen quenching and radical scavenging through both physical and chemical mechanisms [17,18].

When carotenoids absorb visible light, an electron transition from the singlet ground (S_0_) state to the optically allowed second excited singlet (S_2_) state occurs. The S_2_ state typically relaxes to the first excited singlet (S_1_) state before returning to S_0_ state. In carotenoids with a polyene backbone exhibiting C_2h_ point symmetry, the S_0_, S_1_, and S_2_ states correspond to 1^1^A_g_^−^, 2^1^A_g_^−^, and 1^1^B_u_^+^ states, respectively [19]. The transition from S_0_ to S_1_ is forbidden in one-photon optical transitions, making direct excitation unlikely, whereas the S_0_ to S_2_ transition is symmetry-allowed [20]. The singlet excited-state lifetime of carotenoids ranges from hundreds of femtoseconds to a few tens of picoseconds. Femtosecond time-resolved spectroscopy, widely used to monitor these ultrafast relaxation processes, has revealed that energy returns to the S_0_ state via intermediate states like S_X_, S*, and intra-molecular charge transfer (ICT) states during internal conversion and vibrational relaxation [21,22,23,24].

Figure S1B shows the absorption spectrum of the carotenoid spheroidene used in this study, measured at room temperature in THF. The absorption band between 400 and 530 nm corresponds to the S_0_→S_2_ transition and consists of three sub-bands representing the vibrational structure of the S_2_ state. The lowest energy peak, known as the 0-0 transition band, indicates the S_2_ energy level. The energy levels of the S_2_ state shift with the number of conjugated double bonds (*n*), with longer conjugation systems shifting absorption maxima to longer wavelengths [25]. In protein environments, carotenoids typically show red-shifted absorption maxima. In *Rba. sphaeroides*, LH2 complex formation relies on carotenoids from the spheroidene pathway, including neurosporene (*n* = 9), spheroidene (*n* = 10), and spheroidenone (*n* = 10 + C=O), which have extended conjugation systems affecting their spectral properties [25]. In the wild-type strain (*Rba. sphaeroides* 2.4.1), spheroidene is the primary carotenoid.

Chlorophyll, with its extensive cyclic π-electron system, absorbs light across a broad range from near-ultraviolet to near-infrared, enabling efficient sunlight capture for photosynthesis. Porphyrin compounds like chlorophyll, characterized by π-conjugated tetrapyrrole structures, exhibit two main absorption bands: the Q band and the B (Soret) band. These bands are further split into sub-bands (Q_y_, Q_x_, B_y_, and B_x_) due to molecular asymmetry [26,27]. Bchl *a*, a related pigment with a bacteriochlorin ring from the reduction of the 7-8 double bond, displays strong Q_y_ and Soret bands along with a smaller Q_x_ band when well-dispersed as a monomer in polar organic solvents (see Figure S1B).

Excitation energy transfer (EET) between carotenoids and Bchl occurs bidirectionally, but only the transfer from carotenoids to Bchl significantly contributes to light harvesting due to the short excitation lifetime of carotenoids. The internal conversion rates between the S_2_ and S_1_ states of carotenoids depend on their π-electron conjugation length (number of conjugated double bonds, *n*). The S_2_→S_1_ transition occurs within 10–100 fs, while the S_1_→S_0_ transition lasts 10–100 ps, far shorter than the nanosecond-scale S_1_ state lifetime of Bchl [27]. Energy transferred back from Bchl to carotenoids is quickly dissipated as heat via internal conversion during the S_1_→S_0_ transition, preventing energy accumulation and providing photoprotection by dissipating surplus energy transferred from Bchl [28]. Overexcitation of the photosynthetic system generates triplet excited (T_1_) states of Bchl *a*, increasing the risk of singlet oxygen production, a reactive species that causes photodamage. Carotenoids counteract this by accepting triplet energy from T_1_ Bchl *a* via triplet–triplet energy transfer, elevating carotenoids to their own T_1_ state. In this state, they safely dissipate excess energy as heat, protecting the system from photodamage [29].

Since only EET from carotenoids to Bchl contributes to light harvesting, carotenoids must first absorb light to function as antenna pigments. Typically, excitation occurs via the S_0_→S_2_ transition, as the S_0_→S_1_ transition is optically forbidden. The primary transfer pathway is from the S_2_ state of carotenoids to the S_2_ (Q_x_) state of Bchl (S_2_→Q_x_). If the S_2_→S_1_ internal conversion in carotenoids is rapid, transfer from the S_1_ state of carotenoids to the S_1_ state of Bchl (S_1_→Q_y_) can also occur under certain conditions. The close spatial arrangement of carotenoids and Bchl in pigment–protein complexes is crucial for efficient energy transfer. Previous studies have shown that the EET efficiency from carotenoids to Bchl *a* in purple photosynthetic bacteria varies widely (30–90%) depending on the species [28,30,31,32,33,34,35,36,37,38]. However, the mechanisms underlying this transfer, particularly the role of B800 Bchl *a* in LH2 complexes, remain unclear [39]. In this study, photosynthetic membranes of the carotenoid-deficient mutant *Rba. sphaeroides* R26.1 were reconstituted with spheroidene and B800 Bchl *a* to produce reconstituted LH2 complexes. Using advanced spectroscopy, including femtosecond time-resolved absorption, we investigated the energy transfer dynamics from carotenoids to Bchl *a*, focusing on their roles in photoprotection and light harvesting to enhance our understanding of these processes.

## 2. Results

### 2.1. Steady-State Absorption Spectra of Reconstituted LH2 Complexes

**Figure 1:**
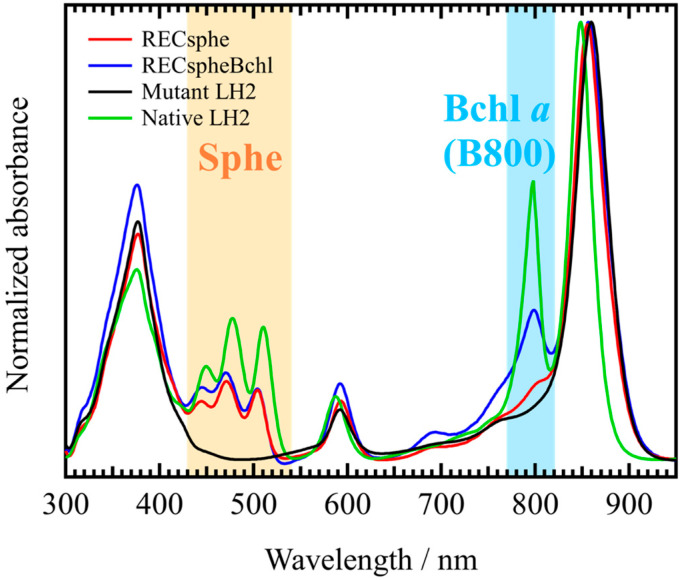
The comparison of steady-state absorption spectra of the LH2 complexes used in this study is shown. The red and blue spectra represent the reconstituted LH2 complexes, RECsphe and RECspheBchl, respectively. The black and green spectra correspond to those of *Rhodobacter sphaeroides* strain R26.1 (Mutant, before reconstitution) and strain 2.4.1 (Native), respectively. To emphasize the spectral region of Car (spheroidene) and B800 Bchl *a,* each region is colored with orange and light blue, respectively.

**Figure 2:**
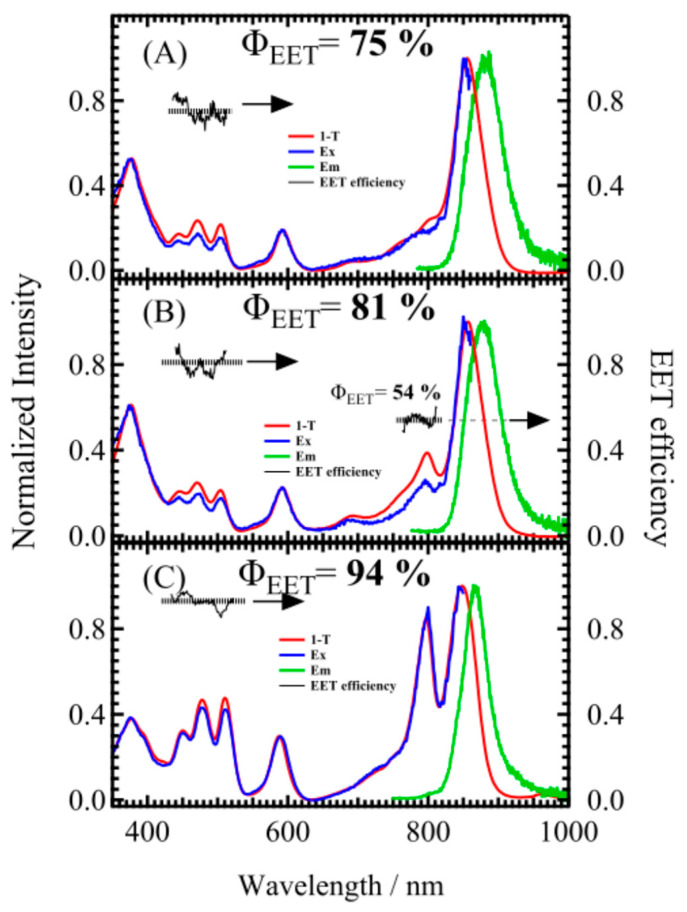
The fractional absorption spectra (red), fluorescence spectra (green), and fluorescence excitation spectra (blue) of LH2 complexes are shown for (**A**) RECsphe, (**B**) RECspheBchl, and (**C**) Native LH2. The inset in each panel displays the EET efficiency from Car (spheroidene) to Bchl a, and from B800 to B850 Bchl a in the case of RECspheBchl. Thin solid black lines represent the experimental results, while thick dashed black lines indicate the average values.

Figure 1 presents the steady-state absorption spectra of the wild-type LH2 complex (Native LH2), the LH2 complex from *Rba. sphaeroides* strain R26.1 (Mutant LH2) before reconstitution with spheroidene and B800 Bchl *a*, and the LH2 complexes after reconstitution. Hereafter, the reconstituted LH2 complex containing only spheroidene is referred to as RECsphe, while the one containing both spheroidene and B800 Bchl *a* is referred to as RECspheBchl. All spectra were normalized to the absorption maximum (~860 nm) of the B850 Bchl *a* Q_y_ band. An absorption peak attributed to the S_0_→S_2_ transition of carotenoids appears between 430 and 540 nm, while another peak around 800 nm corresponds to the monomeric Bchl *a* in the LH2 complex. The absorption wavelengths of the 0-0 transition of carotenoids in the reconstituted LH2 complex are red-shifted by approximately 15 nm compared to those of carotenoids dissolved in solvent (THF). Additionally, the absorption maximum of the Q_y_ state of Bchl *a* in acetone, which is around 770 nm (Figure S1B), also exhibits a shift, further confirming the successful reconstitution of Bchl *a*. These results indicate that both spheroidene and Bchl *a* were effectively reconstituted into the LH2 complex. We believe that the slight absorption band observed in the 800 nm wavelength region of RECsphe LH2 is attributable to free Bchl *a* that has dissociated. This attribution is supported by the absence of a corresponding peak in the fluorescence excitation spectrum (Figure 2A, blue solid line), which validates this interpretation.

### 2.2. Comparison of the Fluorescence and Fluorescence Excitation Spectra of Reconstituted LH2 Complex According to the Existence of B800 Bchl a

To confirm the EET from carotenoids to Bchl *a* within the reconstituted LH2 complex, fluorescence and fluorescence excitation spectra were measured. Figure 2A displays the fluorescence spectrum of RECsphe (green line) alongside the fluorescence excitation spectrum (blue line) detected at 870 nm. The absorption spectrum (red line) is represented on the vertical axis as fractional absorbance (1–T), where T represents transmittance. All measurements were performed at room temperature. All spectra were normalized to the absorption maximum of the Bchl *a* Q_y_ band. The EET efficiency, determined from the ratio of the fluorescence excitation spectrum and fractional absorbance, is shown as a broken black line. The average EET efficiency in the carotenoid absorption region was calculated to determine the excitation energy transfer efficiency from carotenoids to Bchl *a*. Figure 2 also shows the corresponding spectra for (B) RECspheBchl and (C) Native LH2 from the wild-type *Rba. sphaeroides* strain 2.4.1.

### 2.3. Transient Absorption Spectra of Reconstituted LH2 Complexes

**Figure 3:**
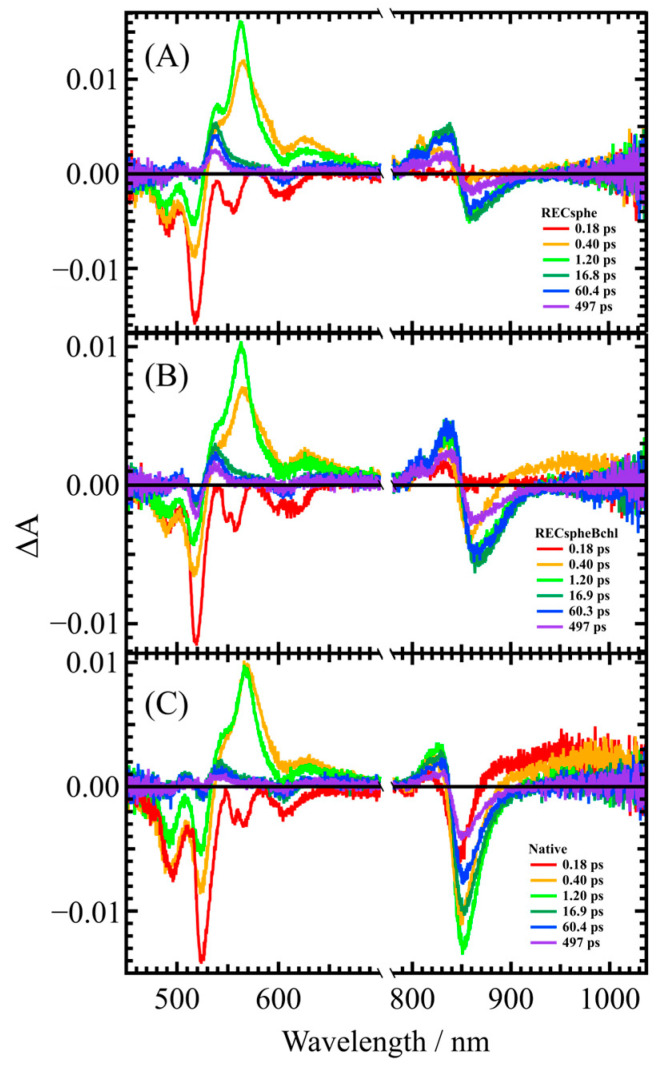
Transient absorption spectra at selected delay times for LH2 complexes of (**A**) RECsphe, (**B**) RECspheBchl, and (**C**) Native LH2 from *Rba. sphaeroides* strain 2.4.1. The spectra illustrate the rise and relaxation of transient absorption signals at different time points.

Figure 3 shows the transient absorption spectra of LH2 complexes, (A) RECsphe, (B) RECspheBchl, and (C) Native, measured using femtosecond time-resolved absorption spectroscopy. These figures present spectra extracted at selected delay times, illustrating the rise and relaxation of transient absorption.

A distinctive feature of the transient absorption spectra of LH2 complexes is a peak around 550 nm, which is attributed to the transient absorption of the carotenoid’s S_1_→S_n_ transition. Here, the S_1_ state represents the first excited singlet state, while the S_n_ state corresponds to an undefined higher excited singlet state. The bleaching observed near 590 nm at approximately 0.18 ps (red line) indicates ultrafast excitation energy transfer from the S_2_ state of the carotenoid to the Q_x_ state of Bchl *a*. In the near-infrared region, the transient absorption signal associated with the S_1_→S_n_ transition of Bchl *a*’s Q_y_ band appears around 830 nm. Additionally, the shift in the negative peak near 860 nm over time indicates that the excitation of the carotenoid gradually alters its surrounding electrostatic environment.

### 2.4. Global Analysis of the Transient Absorption Spectra of Reconstituted LH2 Complexes

Since determining the absorption wavelengths and intensities of excited-state species solely from experimental transient absorption spectra is challenging, global analysis of the entire observed dataset was performed. As a first step, we performed a global analysis using a sequential model. This analysis involved the following steps applied to the full set of transient absorption spectra to derive the Evolution Associated Difference Spectra (EADS) [40,41]. Firstly, transient absorption spectra in the near-infrared region (730–1050 nm) were analyzed to determine the lifetime of the carotenoid’s S_2_ state. This was accomplished using a three-component sequential model in the global analysis. Based on the results of the near-infrared analysis, a global analysis of the visible region (450–700 nm) was conducted, showing that the data could be reasonably interpreted with four or five components. Finally, the same sequential model was applied to the combined data from both the visible and near-infrared regions, integrating information from the entire spectral range.

**Figure 4:**
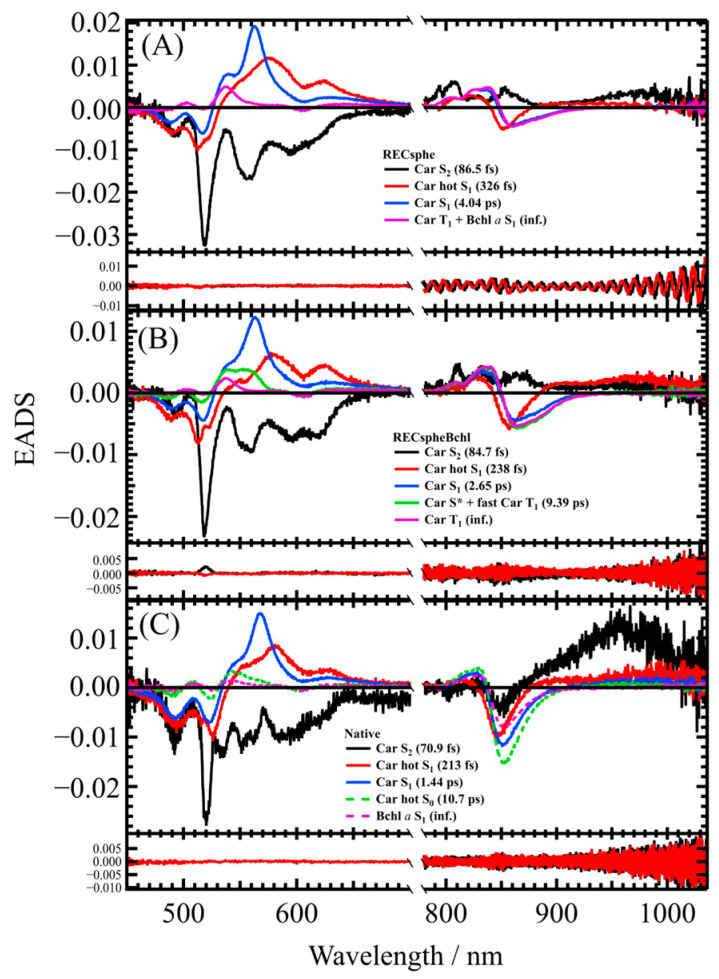
The EADS spectra and the residuals of the LH2 complex obtained from global analysis using a sequential model. The upper row represents RECsphe (**A**), the middle row represents RECspheBchl (**B**), and the lower row represents Native LH2 (**C**). The inset legend shows the assignment and lifetime of each EADS component. The first (solid black line) and second (solid red line) right singular value vectors of the residual matrix, which correspond to the residuals of the fittings, are shown at the bottom of each figure.

Figure 4 presents the EADS derived from global analysis using a sequential model for both the near-infrared and visible regions. The upper row corresponds to RECsphe, the middle row to RECspheBchl, and the lower row to Native LH2. The first EADS component (solid black lines) is attributed to the carotenoid (Car) S_2_ state. In Native LH2, a strong S_2_→S_m_ absorption (where S_m_ represents an undefined higher excited singlet state) is observed near 950 nm. Similarly, in the reconstituted samples, a weaker but comparable absorption is detected, indicating the presence of the same transition. The second (solid red lines) and third (solid blue lines) EADS components are attributed to the vibrationally hot Car S_1_ (Car hot S_1_) state and the relaxed Car S_1_ (Car S_1_) state, respectively. In the near-infrared spectral region, transient absorption and bleaching signals of Bchl *a* are also observed, suggesting EET to Bchl *a* from both excited states of Car. The fourth EADS component (solid magenta line) of RECsphe (Figure 4A) reflects contributions from both the T_1_ state of carotenoid and the S_1_ state of Bchl *a*, based on the spectral band shape and the lifetime (see Figure S3 in Supplementary Materials). This observation suggests the rapid generation of the Car T_1_ state.

On the contrary, the fourth EADS component (solid green line) in RECspheBchl (Figure 4B) is assigned to the Car S* state and the Car T_1_ state, while the fifth EADS component (solid magenta line) is attributed to the Car T_1_ state, as determined by their spectral characteristics. The rapid formation of the Car T_1_ state is attributed to pathways involving either the S* state or a direct transition from the S_2_ state, mediated by singlet–singlet homo-fission within the carotenoid or singlet–singlet hetero-fission involving the S_1_ state of Bchl *a*. These assignments align with previous reports [42,43]. In the fourth and fifth EADS components, transient absorption and bleaching signals of Bchl *a* are also observed.

Notably, the Car S* state was absent in both the RECsphe LH2 and Native LH2 complexes. The fourth EADS component (broken green line) in Native LH2 (Figure 4C) corresponds to the hot S_0_ state of the carotenoid, as inferred from its spectral shape and lifetime (10.7 ps). Additionally, transient absorption and bleaching signals of Bchl *a* are also observed in the fourth EADS component of Native LH2. It should also be noted that the fifth EADS of Native LH2 can be attributed solely to the S_1_ state of Bchl *a*, with no involvement of the Car T_1_ state, which aligns with previous observations [44]. This conclusion is supported by a comparison of spectral band shapes in the visible region, as shown in Figure S3 of the Supplementary Materials.

**Figure 5:**
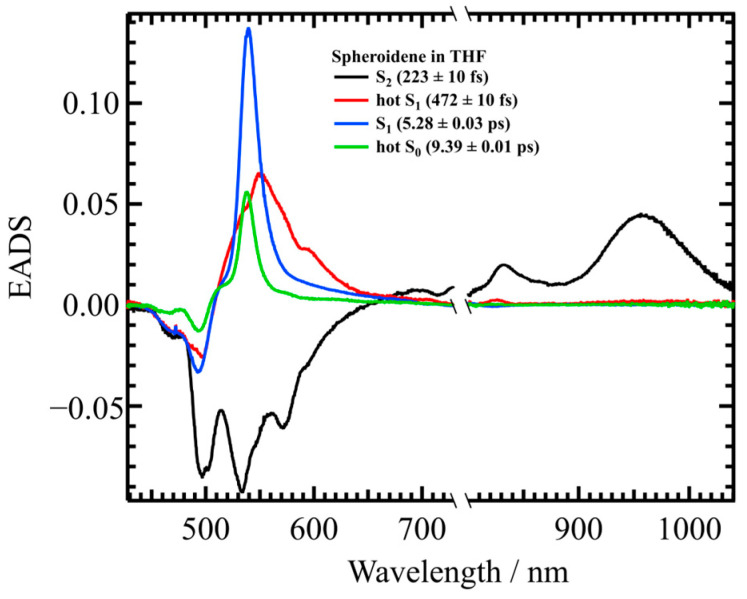
The EADS of spheroidene in THF obtained from global analysis using a four-component sequential model. The inset legend shows the assignment and lifetime of each EADS component.

**Figure 6:**
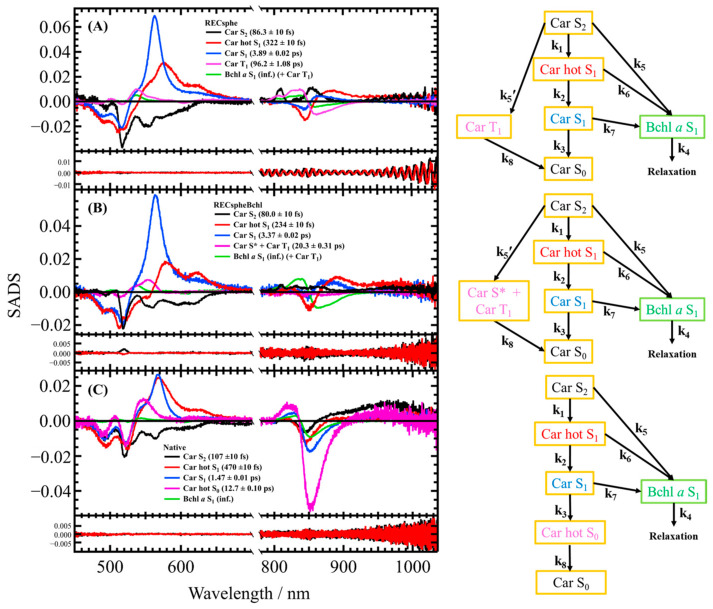
The SADS spectra and the residuals of the LH2 complex obtained from global analysis using a target model. The upper row represents RECsphe (**A**), the middle row represents RECspheBchl (**B**), and the lower row represents Native LH2 (**C**). The right panel of each figure illustrates the analysis model used in the target analysis, with the rate constant for each pathway provided in Table 2 shown in Section 3.3. The first (solid black line) and second (solid red line) right singular value vectors of the residual matrix, which correspond to the residuals of the fittings, are shown at the bottom of each figure. The inset legend shows the assignment and lifetime of each SADS component.

For the further analysis shown in Section 3, it is essential to determine the lifetimes of the excited carotenoid species independent of EET to Bchl *a*. To achieve this, transient absorption spectra of spheroidene in tetrahydrofuran (THF) were analyzed. Figure 5 shows the EADS results for spheroidene in THF, obtained using a four-component sequential model. Based on these results, a more refined target model, as illustrated in each panel of Figure 6, is developed.

## 3. Discussion

### 3.1. Steady-State Spectroscopies of the Reconstituted LH2 Complexes

Based on the absorption spectra shown in Figure 1, we can estimate the extent to which carotenoids and Bchls are reconstituted into the LH2 complexes. By comparing the absorption maximum in the carotenoid region, the absorption of RECsphe and RECspheBchl is approximately 53% of that observed in the Native LH2 complex. This suggests that an average of about five carotenoid molecules are reconstituted per LH2 complex. A similar comparison in the B800 Bchl a region revealed that the absorption of RECspheBchl is also approximately 53% of the native complex. The consistent reconstitution ratios of carotenoids and Bchls imply the possibility that B800 Bchl a may preferentially reconstitute at sites where carotenoids are present. However, this remains a hypothesis that requires further investigation to confirm.

Table 1 summarizes the EET efficiency from spheroidene to Bchl a in the reconstituted LH2 complexes, both with and without B800 Bchl a. The reconstituted LH2 complexes with spheroidene exhibit higher EET efficiency from spheroidene to Bchl a when B800 Bchl a is also reconstituted, as observed in both RECspheBchl and Native LH2. Interestingly, although the carotenoid region absorption is nearly identical between RECsphe and RECspheBchl, as shown in Figure 2, the EET efficiency is notably higher in RECspheBchl. These findings suggest that the presence of B800 Bchl a may play a significant role in enhancing the EET efficiency from carotenoids to Bchl a, as seen in the previous work [39]. It is noteworthy that the EET efficiency from B800 Bchl a to B850 Bchl a in RECspheBchl is only 54%, indicating that only about half of the B800 Bchl a molecules effectively participate in EET. This value, however, may be an overestimate because the absorption region of B800 overlaps with the higher excitonic band of B850, potentially affecting the accuracy of the measurement. Despite this limitation, the EET efficiency from spheroidene to Bchl a in Native LH2 aligns well with previously reported values [45]. Furthermore, a comparison of the full width at half maximum (FWHM) of the fluorescence spectra reveals a decrease in FWHM when B800 Bchl a is present (see Figure S2 and Table S1 in Supplementary Materials). This observation suggests that the presence of B800 Bchl a contributes to a more rigid structural arrangement of the LH2 complex, potentially stabilizing the complex and enhancing its light-harvesting efficiency.

### 3.2. Target Analysis of the Transient Absorption Spectra of Reconstituted LH2 Complexes

Target analysis was conducted to further interpret the transient absorption spectra. This approach enabled a more detailed investigation of the EET processes and excitation energy relaxation pathways. The need for target analysis arose to resolve inconsistencies and address unresolved issues identified in the global analysis described in Section 2.4. While the fitting results shown in Figure 4 are generally satisfactory, some unexplained phenomena have been identified. These include variations in the spectral shape of the Car S_1_ state across different samples and instability in the presence or absence of the Car S* and/or Car T_1_ states between samples. These data inconsistencies highlight the limitations of the global analysis using a sequential model and emphasize the necessity of a more detailed and quantitative approach through target analysis based on a parallel model. Moreover, to elucidate the pathways through which the Car T_1_ state is generated, the construction of a comprehensive model via target analysis is essential. This approach will provide a deeper understanding of the mechanisms underlying the excitation energy dynamics and relaxation pathways.

Figure 6 shows the results of target analysis. The results derived from the target model for RECsphe LH2 (Figure 6A) show that the first Species Associated Difference Spectra (SADS) component (solid black line) represents the relaxation from the Car S_2_ state and EET to Bchl *a*. While EET from the Car S_2_ state to the Bchl *a* S_2_ (Q_x_ state) is theoretically expected, the lifetime of the Bchl *a* S_2_ state is too short (less than 50 fs [46]) to be resolved in this study. Consequently, the target model assumes that excitation energy from the Car S_2_ state is transferred directly to the Bchl *a* S_1_ state. The second SADS component (solid red line) reflects the vibrational relaxation of the Car S_1_ state (Car hot S_1_ state), while the third SADS component (solid blue line) describes relaxation from the Car S_1_ state. To achieve a successful fit, EET pathways from both the Car hot S_1_ and Car S_1_ states to the Bchl *a* S_1_ state were incorporated into the model. The fourth SADS component (solid magenta line) represents the generation of the Car T_1_ component (fast Car T_1_ component) via the Car S_2_ state, as inferred from the EADS of RECsphe LH2 shown in Figure 4A. In a previous study, the Car S* state was identified as a precursor to the rapid generation of the Car T_1_ state [43]. Notably, in the fourth SADS of RECsphe LH2, there is clear evidence of Car T_1_ state production, although the Car S* state is scarcely observed in this LH2 complex (see Figure S4 in Supplementary Materials). The near-infrared region of the fifth SADS component (solid green line) is attributed to the Bchl *a* S_1_ state. Interestingly, a contribution from the Car T_1_ state signal is also observed in the visible region of the fifth SADS (see Figure S5 in Supplementary Materials). This observation suggests the presence of an alternative rapid pathway for generating the Car T_1_ state, occurring on the same time scale as the relaxation of the Bchl *a* S_1_ state. This is supported by the fast Car T_1_ component observed in the fourth SADS, which decays with a lifetime of 96.2 ps. A similar mechanism has been previously reported in the LH1 complex of *Rhodospirillum rubrum* strain S1 [47].

The results for RECspheBchl LH2 (Figure 6B) can be effectively explained using a target model similar to that of RECsphe LH2, with the notable incorporation of the S* state. The first (solid black line), second (solid red line), and third (solid blue line) SADS components correspond to the Car S_2_, Car hot S_1_, and Car S_1_ states, respectively, with EET to the Bchl *a* S_1_ state occurring from each of these states. The fourth SADS component (solid magenta line) originates from the Car S_2_ state; however, its spectral features in the visible region differ significantly from those of RECsphe LH2 (see Figure S4 in the Supplementary Materials). In RECspheBchl LH2, an absorption band is observed around 560 nm, which is notably red-shifted compared to RECsphe LH2, strongly suggesting the presence of the S* state. Additionally, the visible-region spectrum of the fourth SADS in RECspheBchl LH2 exhibits a shoulder absorption band on the shorter wavelength side of the main absorption band, corresponding to the Car T_1_ absorption band. This observation further supports the presence of the S* state as a precursor to the Car T_1_ state. However, it should be noted that these two components cannot be further resolved in this analysis due to the concomitant generation of the Car T_1_ state via an alternative pathway, as discussed below. The near-infrared region of the fifth SADS component (solid green line) is attributed to the Bchl *a* S_1_ state. In the visible region of the fifth SADS, a distinct contribution from the Car T_1_ state is observed. This Car T_1_ state may be generated via the S* state or through an alternative rapid pathway for Car T_1_ generation, as observed for RECsphe LH2. This alternative pathway occurs on the same timescale as the relaxation of the Bchl *a* S_1_ state, similarly to the behavior observed in RECsphe LH2.

The results for Native LH2 are fully explained using the simplest target model, as illustrated in Figure 6C. Interestingly, neither the Car S* state nor the Car T_1_ state was observed in the Native LH2 complexes, which is consistent with a previous report [44]. The first three SADS components (solid black, red, and blue lines) correspond to the Car S_2_, Car hot S_1_, and Car S_1_ states, respectively, and all contribute to EET directed toward the Bchl *a* S_1_ state. In Native LH2, the production of the Car hot S_0_ state during relaxation from the Car S_1_ state was observed, as exemplified in the EADS shown in Figure 4C. The spectral band shape in the visible region of the fourth SADS component further corroborates this observation (see Figure S4 in Supplementary Materials). The fifth SADS component (solid green line) represents a contribution solely from Bchl *a* S_1_. The weakly structured spectral feature in the visible region of the fifth SADS is attributed to the electrostatic effect of Bchl *a* S_1_ on the carotenoid absorption region (dynamic Stark effect) and not to the generation of the Car T_1_ state, as inferred from the spectral band shape (see Figure S5 in Supplementary Materials).

### 3.3. The EET Efficiency from Spheroidene to Bchl a as Determined by Fs Time-Resolved Absorption Spectroscopy

The EET efficiency from spheroidene to Bchl *a* was calculated based on the lifetimes obtained from the target analysis. The calculation of the EET efficiency used the following formula:

$$\emptyset_{ET}=\frac{k_{ET}}{k_{ET}+k_{IC}}\times 100$$

where *k_ET_* is the rate constant for energy transfer from spheroidene to Bchl *a* in the LH2 complex, and *k_IC_* is the rate constant for relaxation of spheroidene in the LH2 complex. Since the fluorescence of carotenoid is known to be very weak, the radiative decay rate is not considered in this formula.

Based on the lifetimes presented in Figure 5 and Figure 6 and Table 2, the EET efficiencies from Car to Bchl *a* were calculated using the formula described above, with the results summarized in Table 3. A comparison of EET efficiencies among the LH2 complexes reveals that Native LH2, in which all carotenoids and B800 Bchl *a* are fully incorporated, exhibits a higher proportion of energy transfer from the Car S_2_ state to Bchl *a* compared to the other two LH2 complexes, where pigments are not fully incorporated. Notably, the contribution of the Car hot S_1_ state to EET is negligibly small in Native LH2, whereas significant EET from the Car S_1_ state is observed. Furthermore, Native LH2 demonstrates the highest overall EET efficiency (86.8%) among the studied LH2 complexes, whereas RECsphe LH2 exhibits the lowest efficiency (50.0%), and RECspheBchl LH2 shows an intermediate efficiency (56.6%). These efficiency values differ from those determined using fluorescence excitation spectroscopy; however, the order of efficiencies remains consistent. The discrepancy in values arises because the *k*_IC_ values were referenced from data obtained in THF solutions, which differ from the actual *k*_IC_ values in the protein environment [40]. Given that the amounts of carotenoids reconstituted into RECsphe LH2 and RECspheBchl LH2 are similar, this observation suggests that the presence of B800 Bchl *a* in RECspheBchl LH2 substantially enhances the EET efficiency from carotenoids to Bchl *a*. The exceptionally high EET efficiency observed in Native LH2, where B800 Bchl *a* is fully incorporated, further supports this conclusion. Additionally, the inclusion of EET pathways from the Car hot S_1_ and Car S_1_ states to Bchl *a* plays a substantial role in increasing the EET efficiencies of RECsphe LH2 and RECspheBchl LH2. These findings underscore the critical influence of B800 Bchl *a* on the efficiency of EET from carotenoid excited states to Bchl *a* in LH2 complexes.

**Table 2:** The rate constant obtained from the target analysis. The analysis model is shown in the right panel of Figure 6.

Rate Constant	RECsphe	RECspheBchl	Native
k_1_	(223 ± 10 fs)^−1^		
k_2_	(472 ± 10 fs)^−1^		
k_3_	(5.48 ± 0.04 ps)^−1^		
k_4_	Infinity		
k_5_	(282 ± 10 fs)^−1^	(249 ± 10 fs)^−1^	(204 ± 10 fs)^−1^
k_5_′	(282 ± 10 fs)^−1^	(249 ± 10 fs)^−1^	-
k_6_	(1.01 ± 0.03 ps)^−1^	(463 ± 10 fs)^−1^	(116 ± 541 ps)^−1^
k_7_	(14.7 ± 0.21 ps)^−1^	(9.27 ± 0.15 ps)^−1^	(2.03 ± 0.02 ps)^−1^
k_8_	(96.2 ± 1.08 ps)^−1^	(20.3 ± 0.31 ps)^−1^	(12.7 ± 0.10 ps)^−1^

Interestingly, the intensity of the SADS is related to the ratio of carotenoids to B800 Bchl *a* EET. When focusing on the intensity of the S_2_ → S_m_ transition in the near-infrared region of the first SADS (black solid line in Figure 6A–C), the intensity is strongest in Native LH2 (0.011 at 965 nm), followed by RECsphe (0.0063) and RECspheBchl (0.0046), which are nearly identical. As mentioned earlier in the steady-state absorption spectra, only half the number of carotenoids is reconstituted in RECsphe and RECspheBchl complexes. This observation suggests that the intensity of the S_2_ state SADS is proportional to the number of carotenoids present in the LH2 complexes. Additionally, considering the intensity of the S_1_ state SADS of carotenoid (blue solid lines in Figure 6A–C), the order of intensity is RECsphe (0.069 at 570 nm) ≥ RECspheBchl (0.059) > Native LH2 (0.027). The EET efficiency from the Car S_1_ state to Bchl *a* follows the same order as the number of B800 Bchl *a* molecules (see Table 3). This suggests that fewer molecules remain in the S_1_ state, as they quickly transfer excitation energy to Bchl *a*, which reduces the probability of S_1_ → S_n_ transitions. Consequently, the stronger the efficiency of EET to Bchl *a*, the lower the intensity of the S_1_ peak. In other words, excitation energy from the Car S₁ state is likely transferred to B800 Bchl *a* rather than B850 Bchl *a*. However, this hypothesis requires further investigation and more detailed studies for conclusive confirmation.

## 4. Materials and Methods

For each sample, preparation was repeated at least three times, and three independent measurements were conducted on highly consistent samples.

### 4.1. Cultivation of Bacteria

The purple photosynthetic bacteria were cultured in C-succinate media [48]. The media composition has been specified in Supplementary Materials. The media were sterilized at 126 °C for 20 min using an autoclave (ST500 Yamato Scientific Co., Ltd., Tokyo, Japan). Seed cells of *Rhodobacter sphaeroides* strain R26.1 were inoculated into the culture bottles under a clean bench to prevent air contamination. The cultures were incubated photosynthetically for 7 days at 27 °C under anaerobic conditions in a bioclimatic chamber (KCLP-1400ⅡCT-S, Nippon Medical and Chemical Instruments Co., Ltd., Osaka, Japan), using a 100 W incandescent bulb as the light source. To ensure sufficient anaerobic conditions during the first day of incubation, the culture bottles were shielded from light to deplete oxygen. After incubation, the cultured cells were harvested by centrifugation (CR20G, Eppendorf Himac Technologies Co., Ltd., Ibaraki, Japan) at 18,800× *g* for 30 min at 4 °C. The harvested cells were resuspended in a 20 mM MES buffer (pH 6.8) containing 100 mM KCl and stored frozen at −30 °C until further use.

### 4.2. Preparation of Chromatophores

The chromatophores of *Rhodobacter sphaeroides* strain R26.1 were prepared following the method of Davidson et al. [49]. Harvested cells were homogenized in 20 mM Tris-HCl buffer (pH 8.0) containing a small amount of DNase and anhydrous magnesium chloride. The homogenized cells were subjected to high-pressure disruption (~100 MPa, applied twice) using a French press (5501-M, Ohtake Manufacturing Co., Tokyo, Japan). Unbroken cells and cell walls were removed by centrifugation at 2280× *g* for 10 min at 4 °C, and the supernatant was collected. Chromatophores were further isolated by ultracentrifugation (CR70MX, Eppendorf Himac Technologies Co., Ltd., Ibaraki, Japan) at 183,000× *g* for 90 min at 4 °C. The resulting pellet was resuspended in 20 mM Tris-HCl buffer (pH 8.0). Finally, the chromatophores were freeze-dried using a freeze dryer (FDU-12AS, Tokyo Rikakikai Co., Ltd., Tokyo, Japan) and stored at −30 °C until use.

### 4.3. Isolation and Purification of Pigments

#### 4.3.1. Isolation and Purification of Spheroidene

Spheroidene was isolated from *Rhodobacter sphaeroides* strain 2.4.1 using the method described in reference [50]. The strain was cultured and harvested in the same manner as *Rba. sphaeroides* strain R26.1. Harvested cells were mixed with an acetone/methanol solution (7:2, *v*/*v*) and stirred under a nitrogen atmosphere at room temperature for 10 min to extract the pigments. The mixture was centrifuged at 5000× *g* for 10 min at 4 °C, and the supernatant was collected. This extraction process was repeated three times, with stirring extended to 30 min for subsequent extractions. The supernatants from all extractions were combined and subjected to liquid–liquid separation using *n*-hexane and saturated aqueous sodium chloride. The *n*-hexane layer was further extracted with a methanol/water solution (95:5, *v*/*v*) to remove Bchl *a*. The *n*-hexane layer was then dried over sodium sulfate, and the solvent was removed using a rotary evaporator, yielding crude spheroidene. The crude spheroidene was dissolved in a small amount of *n*-hexane and purified using silica gel column chromatography. The elution solvent was 10% diethyl ether in *n*-hexane, and the fractions containing carotenoids were monitored by absorption spectroscopy.

#### 4.3.2. Isolation and Purification of Bchl *a*

Bchl *a* was isolated from *Rhodobacter sphaeroides* strain R26.1 using the method described in references [51,52]. Harvested cells were freeze-dried and mixed with an acetone/methanol solution (1:1, *v*/*v*), then stirred in an ice bath for 1 h. The extracted pigments were separated by centrifugation at 18,800× *g* for 10 min at 4 °C, and the supernatant was collected. This process was repeated three times under the same conditions. The collected supernatants were concentrated using a rotary evaporator to obtain crude Bchl *a*. The crude Bchl *a* was dissolved in a small amount of *n*-hexane/isopropanol solution (96:4, *v*/*v*) and purified using silica gel column chromatography. The elution solvent was *n*-hexane/isopropanol solution (96:4, *v*/*v*). The purified Bchl *a* was concentrated using a rotary evaporator, dissolved in a small amount of acetone, and stored at −30 °C until use. Bchl exhibits a characteristic Q_y_ band in the near-infrared region, which sometimes undergoes a blue shift (shorter wavelength shift) upon pheophytinization. For instance, the Bchl Q_y_ band (770–800 nm) shifts to the pheophytin Q_y_ band (665–670 nm). During the preparation of reconstituted samples and before and after measurements, we confirmed that no such peak shift occurred by examining steady-state absorption spectra.

### 4.4. Reconstitution Procedure

#### 4.4.1. Reconstituting Carotenoids and Bchl *a* to the Chromatophore of *Rba. sphaeroides* Strain R26.1

The reconstitution experiments were conducted as described in reference [49]. Isolated and purified carotenoids were dissolved in petroleum ether (OD_MAX_ = 10), and 0.25 g of freeze-dried chromatophores was added. The carotenoid-to-Bchl *a* ratio in the chromatophores was adjusted to 5 mol:1 mol. This solution was sonicated for 120 min using an ultrasonic bath (SND, US-KS) to disperse aggregated chromatophores. After sonication, the mixture was concentrated using a rotary evaporator, and 20 mM Tris-HCl buffer (pH 8.0) was added to dissolve the residue. Excess carotenoids were removed by ultracentrifugation at 10,000× *g* for 60 min at 4 °C. The resulting precipitate was resuspended in a small amount of 20 mM Tris-HCl buffer (pH 8.0) and stored at 4 °C until use. For reconstitution with both carotenoids and Bchl *a*, a similar procedure was followed. In this case, both carotenoids and Bchl *a* (OD_MAX_ = 5) were dissolved in petroleum ether. The ratio of Bchl *a* in the solution to Bchl *a* in the chromatophores was adjusted to 5 mol:1 mol.

#### 4.4.2. Isolating and Purifying the LH2 from the Carotenoid Reconstituted Chromatophore Solution

The concentration of the reconstituted chromatophore solution was adjusted with 20 mM Tris-HCl buffer (pH 8.0) to achieve an absorbance of 50 at 850 nm (OD₈₅₀), corresponding to the Q_y_ band of Bchl *a*. To this solution, 4% (*w*/*v*) of the detergent *n*-Decyl-β-D-maltoside (DM) was added, and the mixture was incubated in the dark at room temperature for 60 min to solubilize the chromatophores. Following solubilization, the mixture was centrifuged at 6000× *g* for 10 min at 4 °C to remove unsolubilized material, and the supernatant was collected.

The supernatant was then used to isolate the reconstituted LH2 complex via sucrose density gradient ultracentrifugation. The sucrose density gradient was prepared by incrementally adjusting the sucrose concentration from 1.0 mM to 1.6 mM in steps of 0.1 mM using a solution containing sucrose, 20 mM Tris-HCl buffer (pH 8.0), and 0.15% DM. The solubilized sample solution was loaded onto the gradient, and ultracentrifugation was performed at 148,000× *g* for 14 h at 4 °C. The layer containing the reconstituted LH2 complex was collected.

After ultracentrifugation, the collected LH2 complex was further purified using ion-exchange column chromatography (DE52, Cytiva, Tokyo, Japan). The DE52 column was pre-equilibrated with 20 mM Tris-HCl buffer (pH 8.0) containing 0.15% DM. The NaCl concentration in the eluent was gradually increased from 20 mM to 200 mM using a solution of NaCl, 20 mM Tris-HCl buffer (pH 8.0), and 0.15% DM. Excess carotenoids were eluted with 20 mM NaCl, followed by the reconstituted LH2 complex eluted with 200 mM NaCl. The collected LH2 complex was concentrated using a centrifugal ultrafiltration filter (Amicon Ultra, Merck KGaA, Darmstadt, Germany) and stored for subsequent use.

#### 4.4.3. Isolating and Purifying the LH2 from the Carotenoid and Bchl *a* Reconstituted Chromatophore Solution

The concentration of the reconstituted chromatophore solution was adjusted with 20 mM Tris-HCl buffer (pH 8.0) to achieve an absorbance of 50 at 850 nm, corresponding to the Q_y_ band of Bchl *a*. To this solution, 2% (*w*/*v*) of the surfactant *n*-Dodecyl-β-D-maltoside (DDM) was added, and the mixture was incubated in the dark at room temperature for 60 min to achieve solubilization. After solubilization, the sample was diluted fivefold using 20 mM Tris-HCl buffer (pH 8.0), and the insoluble components were removed by centrifugation at 6000× *g* for 10 min at 4 °C. The supernatant was then used to isolate and purify the reconstituted LH2 complexes via ion-exchange column chromatography with a DE52 column. The eluent consisted of a solution of NaCl, 20 mM Tris-HCl buffer (pH 8.0), and 0.15% DDM. The column was operated as previously described, and the reconstituted LH2 complexes were eluted. The collected LH2 complexes were concentrated using a centrifugal ultrafiltration filter (Amicon Ultra, Merck KGaA, Darmstadt, Germany) and stored for further use.

### 4.5. Spectroscopic Measurements

#### 4.5.1. Steady-State Absorption Spectroscopy

The absorption spectrum was recorded using a double-beam UV-visible-near-infrared spectrophotometer (V-670, JASCO Corporation, Tokyo, Japan) equipped with a 10 mm path length quartz cuvette. The steady-state absorption spectrum was measured at room temperature.

#### 4.5.2. Fluorescence and Fluorescence Excitation Spectroscopy

Fluorescence and fluorescence excitation spectra were recorded using a spectrofluorometer (Duetta, Horiba, Ltd., Kyoto, Japan). For fluorescence spectrum measurements, excitation was performed at wavelengths corresponding to the absorption maxima of each carotenoid. For fluorescence excitation spectrum measurements, emission was detected at 870 nm, corresponding to fluorescence from the Q_y_ absorption band of Bchl *a*. Samples for both fluorescence and fluorescence excitation measurements were prepared with an optical density of approximately 0.3 and measured in a 10 mm path length, four-sided transparent quartz cuvette. All measurements were conducted at room temperature.

#### 4.5.3. Time-Resolved Absorption Spectroscopy

The details of the femtosecond time-resolved absorption spectroscopy system have been outlined in previous works [41]. The light source was a regenerative amplified mode-locked Ti:Sapphire laser pulse (Solstice Ace, Spectra-Physics, Milpitas, CA, USA, central wavelength: 784 nm, pulse repetition frequency: 1 kHz, output: 7 mJ/pulse, pulse width: ~80 fs). The laser pulse was split into pump and probe beams using a beam splitter. The wavelength of the pump beam was converted using an optical parametric amplifier (TOPAS Prime, Spectra-Physics, Milpitas, CA, USA). The 1 kHz probe light pulse output from TOPAS Prime was used to measure the spectrum before and after excitation by the pump light, and the difference in these spectra was used to obtain the transient absorption spectrum. The probe beam was focused onto a 2 mm or 7 mm thick sapphire plate, inducing self-phase modulation to generate a visible white light probe beam. This probe light was spatially aligned with the pump light and irradiated onto the sample. After passing through the sample, the probe beam was directed into a spectrometer and detected by a 1024-channel CMOS linear image sensor (S3903-1024Q, Hamamatsu Photonics K.K., Shizuoka, Japan). The 1 kHz laser repetition frequency served as the timing trigger, while a chopper, operating at 500 Hz, blocked light periodically. The signal readout clock, along with the chopper, was controlled by a synchronization circuit developed in-house. This setup enabled measurement of intensity changes in the probe and reference beams at 1 ms intervals after pump light excitation. Mechanical vibrations and slow fluctuations, such as acoustic noise, were minimized, achieving a noise level of approximately ΔA = 10⁻⁴. The excitation wavelengths used were 507 nm for RECsphe and RECspheBchl and 515 nm for wild-type LH2. Time-resolved spectra for each sample were recorded at room temperature, spanning from −2.0 ps to 500 ps after excitation.

#### 4.5.4. Analysis of Transient Absorption Spectra

The transient absorption spectra obtained from time-resolved absorption spectroscopy contain critical information about excited states, vibrational relaxation, internal conversion, and other processes, making it challenging to identify individual components. To address this, Singular Value Decomposition (SVD) analysis, along with global and target analysis, was performed to evaluate how each component behaves over time, including energy transfer and state transitions. The analysis involves recursive calculations, iterating until the residuals between the experimentally obtained transient absorption spectra and the calculated values—based on excitation–relaxation dynamics inferred from scientific reasoning and the number of components determined by SVD analysis—are minimized. This approach enables the identification of the components present in the time-resolved absorption spectra and the determination of their respective lifetimes. Additionally, convolution with an Instrument Response Function (IRF) and chirp correction was applied to minimize delays in the signals of the measuring instruments and to correct for wavelength-dependent dispersion of the probe light during measurement. The entire process, from SVD analysis to global and target analysis, was conducted using the Glotaran Program [40].

## 5. Conclusions

In this study, spheroidene and Bchl *a* were successfully reconstituted into the chromatophores of the carotenoidless mutant *Rhodobacter sphaeroides* strain R26.1, yielding high-quality LH2 complexes. Comprehensive global and target analyses of transient absorption data provided a robust framework to elucidate the excited-state energy dynamics of each LH2 complex. Using rate constants derived from the target analysis, we calculated the EET efficiencies, revealing that the incorporation of B800 Bchl *a* plays a pivotal role in modulating EET efficiency from each excited state of the carotenoid. Our findings demonstrated that the EET pathways and efficiencies from carotenoid to Bchl *a* are intricately dependent on the pigment composition and ratio within the LH2 complexes. In all the LH2 complexes studied, energy transfer predominantly occurred from the Car S_2_ state, whereas in Native LH2, additional EET pathways from the Car S_1_ state became prominent. Notably, the relaxation pathways differed significantly among RECsphe, RECspheBchl, and Native LH2 complexes. The generation of the Car S* state was exclusively observed in RECspheBchl LH2, attributed to the presence of an intermediate number of B800 Bchl *a* molecules. In contrast, the Car T_1_ state was generated in both reconstituted complexes but not in Native LH2, where carotenoids and Bchl *a* were fully incorporated, albeit without B800 Bchl *a*. These results provide critical insights into the intricate energy transfer mechanisms underpinning photosynthetic light-harvesting systems. They highlight the nuanced interplay between pigment composition and energy dynamics, offering a deeper understanding of how structural variations influence EET efficiency. This study lays a solid foundation for advancing artificial photosynthesis and developing innovative strategies for sustainable energy production, paving the way for future breakthroughs in bio-inspired energy technologies.

## Figures and Tables

**Table 1:** The total excitation energy transfer (EET) efficiency from carotenoids to Bchl *a* in the LH2 complexes used in this study is presented.

	EET Efficiency (%)	
	Car → B850 Bchl	B800 Bchl *a* → B850 Bchl *a*
RECsphe	75 ± 4	N/A
RECspheBchl	81 ± 5	54 ± 4
Native	94 ± 2	100 ± 6

**Table 3:** The EET efficiency from spheroidene to Bchl *a*. The highest EET efficiency for each sample is highlighted in bold. The values in parentheses indicate the overall efficiency.

	S_2_ → Bchl *a* (%)	hot S_1_ → Bchl *a* (%)	S_1_ → Bchl *a* (%)
RECsphe (50.0 ± 1.38)	30.6 ± 0.98	31.8 ± 1.23 (12.3 ± 0.79)	26.4 ± 0.40 (7.04 ± 0.57)
RECspheBchl (56.6 ± 1.48)	32.0 ± 0.87	50.5 ± 0.96 (18.2 ± 0.94)	36.3 ± 0.63 (6.47 ± 0.73)
Native (86.8 ± 1.48)	52.2 ± 0.01	0.41 ± 1.9 (0.20 ± 0.91)	72.2 ± 1.0 (34.4 ± 0.81)

## Data Availability

The data that support the findings of this study are available from the authors on reasonable request.

## Supplementary Materials

The following supporting information can be downloaded at: https://www.mdpi.com/article/10.3390/molecules30040814/s1, Figure S1. (A) The structure of the LH2 complex. Carotenoids, B800 Bchl *a*, and B850 Bchl *a* molecules are depicted using orange, magenta, and blue lines, respectively (PDB ID: 7pbw). (B) The steady-state absorption spectra are illustrated with distinct color fills. Figure S2. (A) Comparison of the fluorescence spectra of the LH2 complexes examined in this study. Each spectrum corresponds to RECsphe (red solid line), RECspheBchl (blue solid line), and Native LH2 (green solid line).(B), (C), and (D) present the Gaussian fitting results for RECsphe, RECspheBchl, and Native LH2, respectively. Figure S3. Comparison of the visible region of the fourth component of the EADS, as presented in Figure 5 of the main manuscript. Figure S4. Comparison of the visible region of the fourth component of the SADS, as presented in Figure 6 of the main manuscript. Figure S5. Comparison of the visible region of the fifth component of the SADS, as presented in Figure 6 of the main manuscript. Table S1. The bandwidths obtained from the Gaussian fitting shown in Figure S1. Table S2. Ingredients for C-succinate media (10 L preparation). Table S3. Ingredients for Concentrated Base. Table S4. Ingredients for Metos44. Table S5. Ingredients for Growth Factors.
