# Supplementary material for: Excitation Energy Transfer Dynamics from Carotenoid to Bacteriochlorophyll a in the LH2 Complex of Rhodobacter sphaeroides: Insights from Reconstitution Experiments with Carotenoids and B800 Bacteriochlorophyll a
Source: Molecules. 2025 Feb 10;30(4):814. doi: 10.3390/molecules30040814 (PMC11858093; doi:10.3390/molecules30040814)
Supplement: Supplementary file 1 [file molecules-30-00814-s001.zip › molecules-3440246-supplementary.pdf]

Supplementary Materials

Excitation Energy Transfer Dynamics from Carotenoids to Bacteriochlorophyll *a* in the LH2 Complex of *Rhodobacter sphaeroides*: Insights from Reconstitution Experiments with Carotenoids and B800 Bacteriochlorophyll *a*

Chiasa Uragami[1], Marina Yoshida[1], Alastair T. Gardiner[2], Richard J. Cogdell[3] and Hideki Hashimoto[1*]

[1]*Kwansei Gakuin University, Department of Applied Chemistry for Environment, Graduate School of Science, 1-Gakuen-Uegahara, Sanda, Hyogo 669-1330, Japan*
[2] *Czech Academy of Sciences, Institute of Microbiology, 379 81 Třeboň, Czech Republic*
[3]*University of Glasgow, School of Molecular Biosciences, Glasgow G12 8QQ, Scotland, UK*

* Corresponding author: Hideki Hashimoto, E-mail: hideki-hassy@kwansei.ac.jp

1. Structure and absorption spectra of LH2 complexes

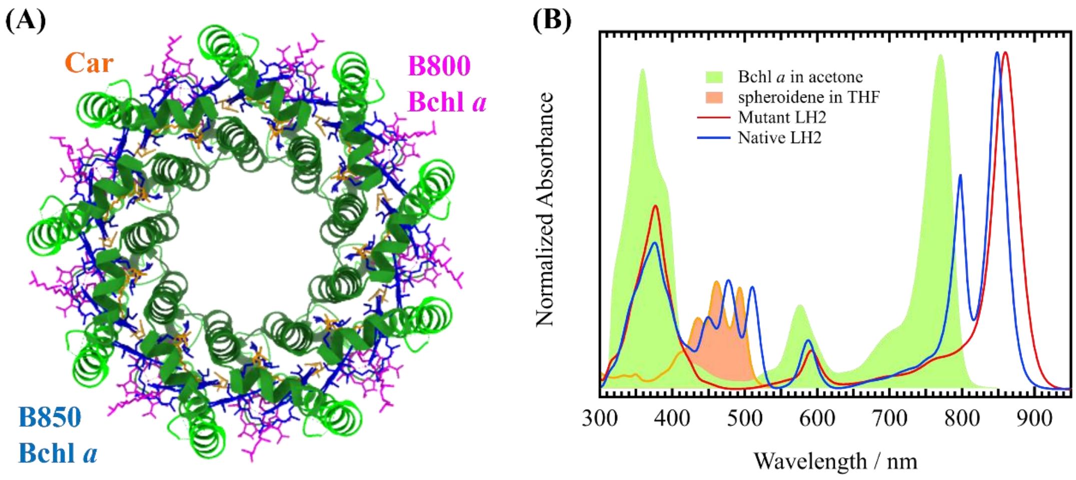

Figure S1. (A) The structure of the LH2 complex. Carotenoids, B800 Bchl *a*, and B850 Bchl *a* molecules are depicted using orange, magenta, and blue lines, respectively (PDB ID: 7pbw). (B) The steady-state absorption spectra are illustrated with distinct color fills. The spectrum with an orange fill represents spheroidene in THF, while the light green fill corresponds to Bchl *a* in acetone. The red solid line shows the absorption spectrum of the LH2 complex from *Rhodobacter sphaeroides* strain R26.1 (a carotenoidless mutant), and the blue solid line represents the spectrum from strain 2.4.1 (a wild type).

2. Comparison of fluorescence spectra of LH2 complexes.

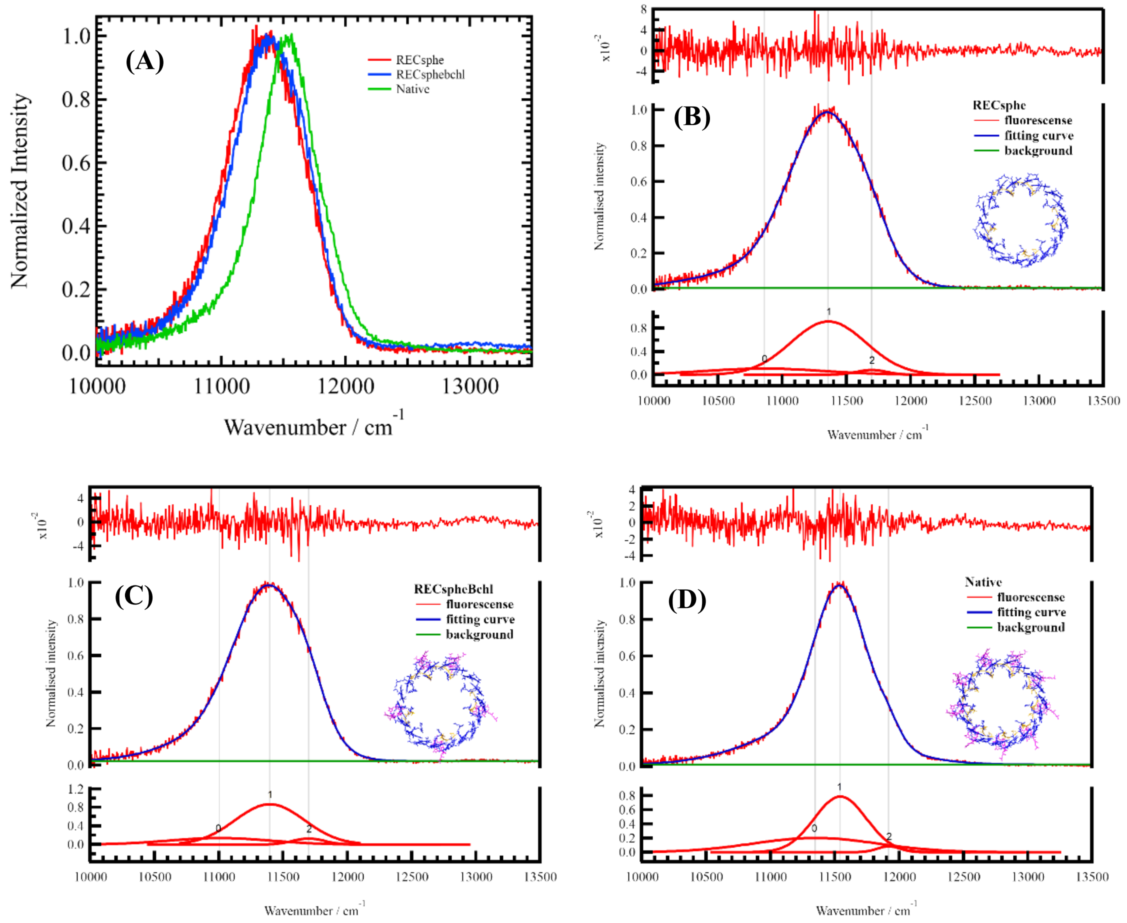

Figure S2. (A) Comparison of the fluorescence spectra of the LH2 complexes examined in this study. Each spectrum corresponds to RECsphe (red solid line), RECspheBchl (blue solid line), and Native LH2 (green solid line).(B), (C), and (D) present the Gaussian fitting results for RECsphe, RECspheBchl, and Native LH2, respectively. For each figure, the upper row displays the residuals of the fitting, the middle row shows the overlap of the fitting curve (blue solid line) and the experimental data (red solid line), and the lower row illustrates the shape of each Gaussian band used for the fitting. All fluorescence bands were fitted with three Gaussian functions. Additionally, the possible structures of each LH2 complex are depicted in panels (B), (C), and (D).

Table S1. The bandwidths obtained from the Gaussian fitting shown in Figure S1. The main band narrows in the order of RECsphe, RECspheBchl, and Native LH2, reflecting the increasing rigidity of the binding of Bchl *a* molecules to the LH2 complexes.

	**Band 0 (cm^{-1})**	**Band 1 Main band (cm^{-1})**	**Band 2 (cm^{-1})**
RECsphe	654.02	411.74	164.57
RECspheBchl	565.66	381.06	178.07
Native	686.14	286.73	129.23

3. **Comparison of the fourth EADS component across reconstituted systems and Native LH2, including the fifth EADS component of Native LH2.**

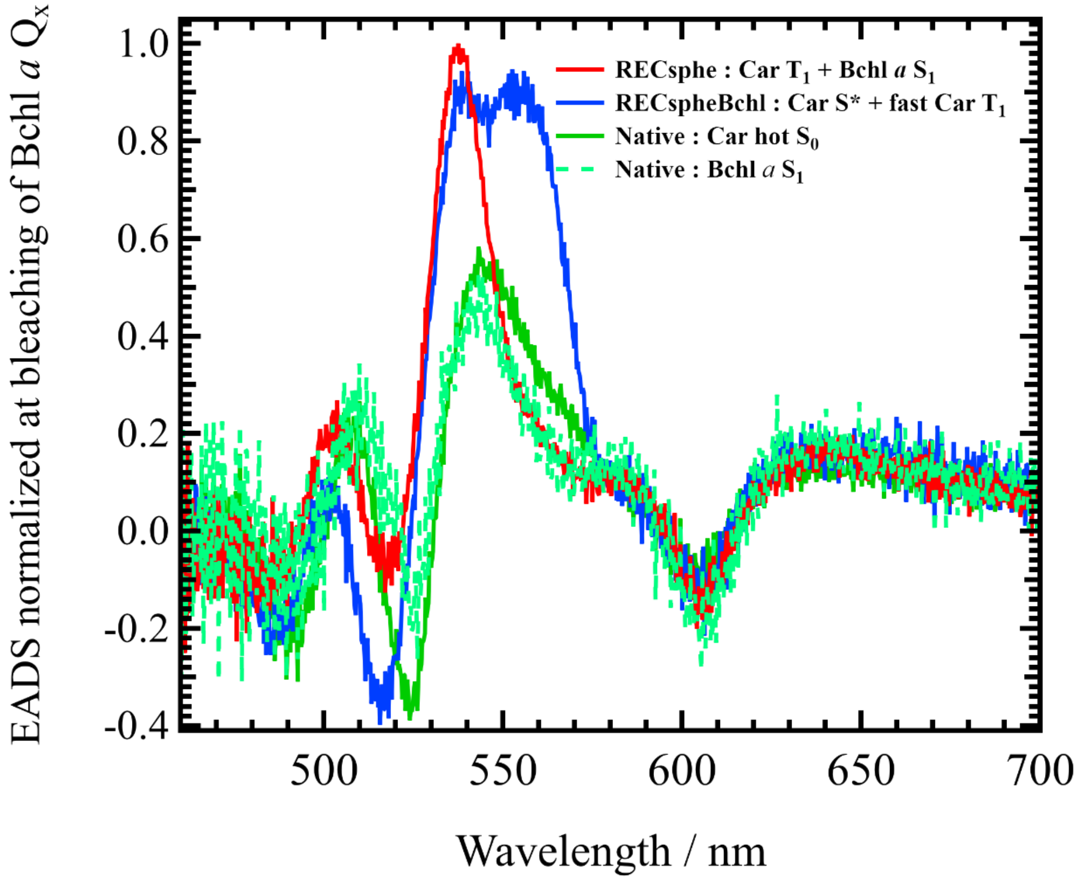

Figure S3. Comparison of the visible region of the fourth component of the EADS, as presented in Figure 5 of the main manuscript. The spectra were normalized to the bleaching signal of the Bchl *a* Q_x band. This comparison reveals a distinct difference in the band shape around 550 nm. The peak observed for RECsphe (solid red line) is interpreted as originating from Car T_1, while the peaks in RECspheBchl (solid blue line) are attributed to Car T_1 and Car S^*. The band observed for Native LH2 (solid green line) is attributed to Car hot S_0. Additionally, the fifth EADS component of Native LH2 (broken green line) is included for a more comprehensive understanding.

4. Comparison of fourth component of SADS.

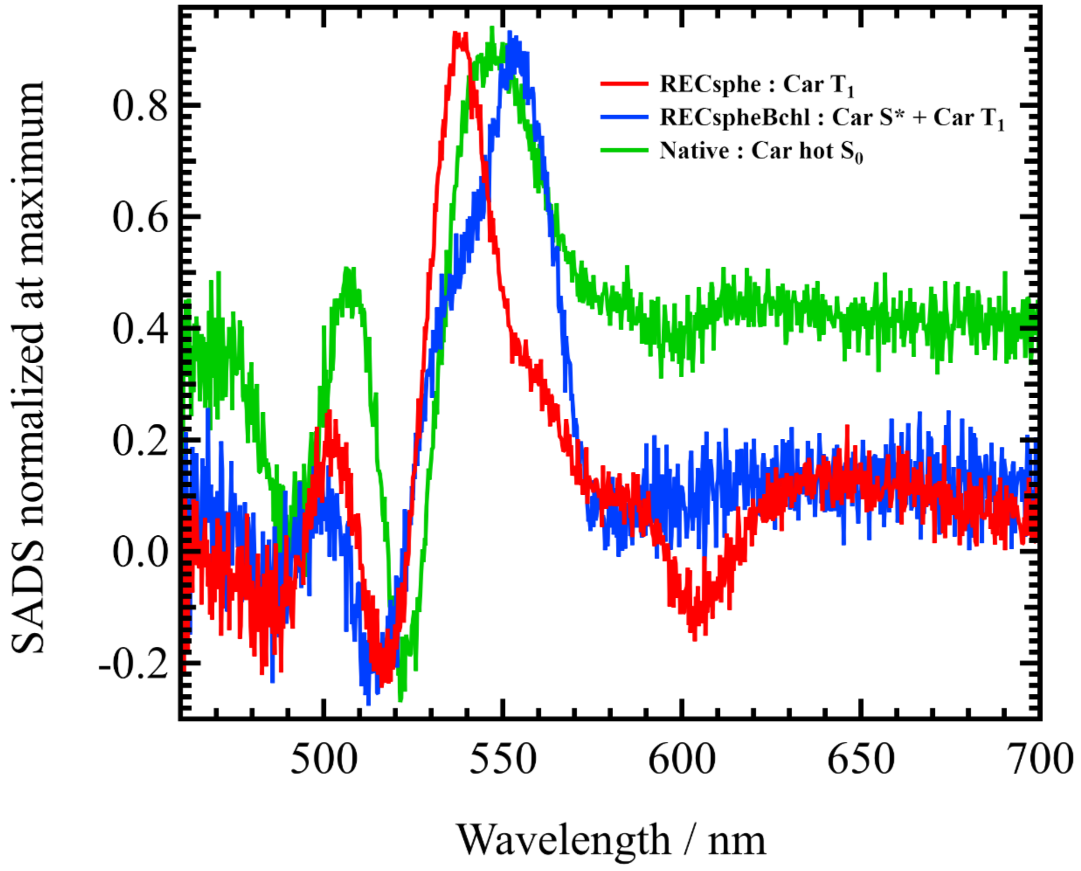

Figure S4. Comparison of the visible region of the fourth component of the SADS, as presented in Figure 7 of the main manuscript. The spectra were normalized to the bleaching signal in the 510–520 nm spectral region. This comparison reveals a distinct difference in the band shape around 550 nm.

5. Comparison of fifth component of SADS.

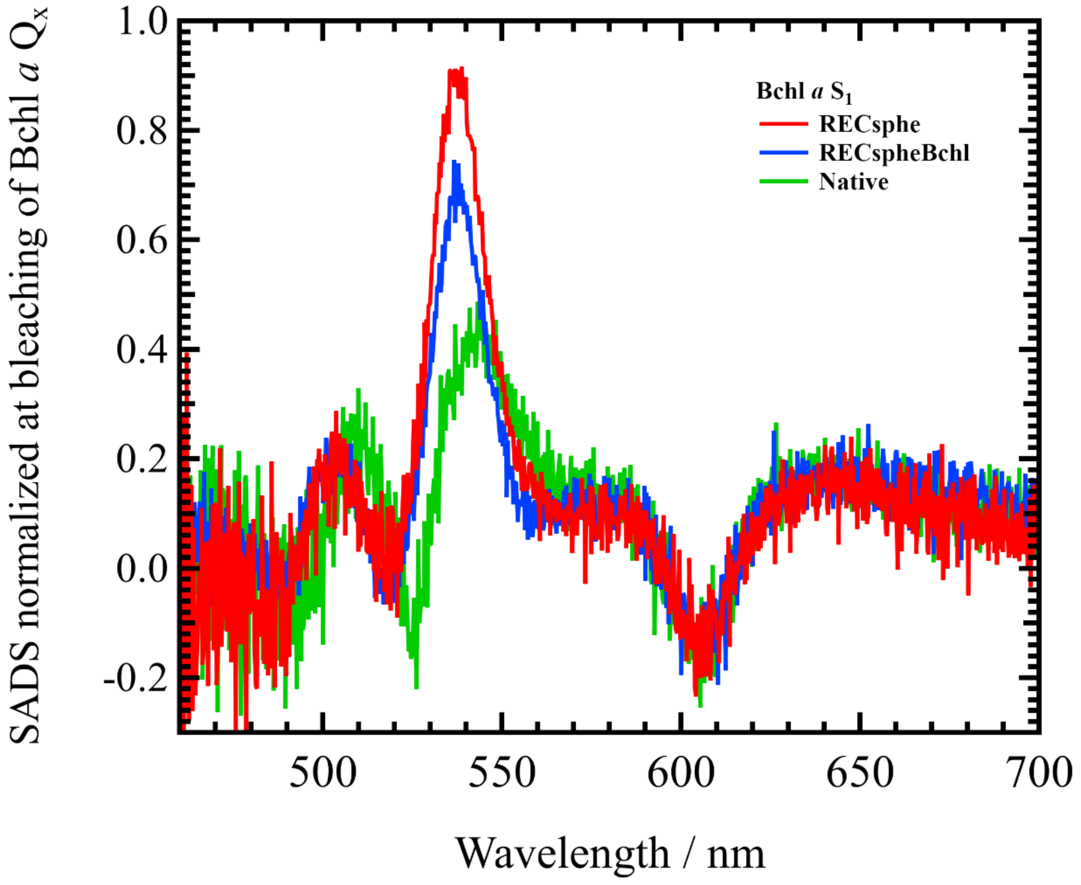

Figure S5. Comparison of the visible region of the fifth component of the SADS, as presented in Figure 7 of the main manuscript. The spectra were normalized to the bleaching signal of the Bchl *a* Q_x band. This comparison highlights a distinct difference in the band shape around 540 nm for Native LH2, whereas RECsphe and RECspheBchl exhibit similar band shapes attributable to Car T_1.

6. The composition of the media.

Purple photosynthetic bacteria were cultured using C-succinate media [48] The C-succinate media was prepared by mixing the reagents listed in Table S2 with distilled water to make 10 L. The prepared media was sterilized at 126°C for 20 minutes using an autoclave (ST500, Yamato Scientific co., ltd., Tokyo, Japan).

Table S2. Ingredients for C-succinate media (10 L preparation)

Concentrated Base (pH6.8)	200 mL
1M Potassium Hydrogen Phosphate	100 mL
1M Potassium Dihydrogen Phosphate	100 mL
10%[w/w] Ammonium Sulfate	50 mL
1M Potassium Succinate (pH6.8)*	100 mL
Growth Factors	10 mL
Casamino Acids	10 g

* Adjust the pH using NaOH.

The Concentrated Base used for preparing C-succinate media was made by mixing the reagents listed in Table S3 with distilled water and adjusting the pH to 6.8 using sodium hydroxide. The final volume was adjusted to 1 L.

Table S3. Ingredients for Concentrated Base

Nitrilotriacetic Acid	10 g
Magnesium Sulfate Heptahydrate	29.58 g
Calcium Chloride Dihydrate	3.40 g
Ammonium Molybdate	9.82 mg
Ferric Sulfate Heptahydrate	99 mg
Nicotinic Acid	50 mg
Thiamine Hydrochloride	25 mg
Biotin (Vitamin H)	5 mg
Metos44	50 mL

Metos44 used for preparing the Concentrated Base was made by mixing the reagents listed in Table S4 with distilled water to a final volume of 1 L.

Table S4. Ingredients for Metos44

Disodium Ethylenediaminetetraacetate (EDTA)	2.5 g
Zinc Sulfate Heptahydrate	19.5 g
Manganese Sulfate Pentahydrate	1.66 g
Copper Sulfate Pentahydrate	0.392 g
Cobalt Sulfate Hexahydrate	0.248 g
Ferric Sulfate Heptahydrate	5.50 g
Sodium Tetraborate Decahydrate	0.177 g
Sulfuric Acid	Few drops

The Growth Factors used for preparing C-succinate media were made by mixing the reagents listed in Table S5 with distilled water to a final volume of 1 L. Initially, biotin and sodium bicarbonate were dissolved in a small amount of distilled water. The remaining reagents were then added, and the solution was heated while adjusting the volume to 1 L with distilled water.

Table S5. Ingredients for Growth Factors

Biotin (Vitamin H)	0.02 g
Sodium Bicarbonate	0.5 g
Nicotinic Acid	1.0 g
Thiamine Hydrochloride	0.5 g
p-Aminobenzoic Acid	1.0 g

^
